# Identification of *Lactobacillus* from the Saliva of Adult Patients with Caries Using Matrix-Assisted Laser Desorption/Ionization Time-Of-Flight Mass Spectrometry

**DOI:** 10.1371/journal.pone.0106185

**Published:** 2014-08-28

**Authors:** Yifei Zhang, Yingyi Liu, Qingwei Ma, Yeqing Song, Qian Zhang, Xiaoyan Wang, Feng Chen

**Affiliations:** 1 Central Laboratory, Peking University School and Hospital of Stomatology, Beijing, P.R. China; 2 Department of Cariology and Endodontology, Peking University School and Hospital of Stomatology, Beijing, P.R. China; 3 Academy for Advanced Interdisciplinary Studies, Peking University, Beijing, China; Moffitt Cancer Center, United States of America

## Abstract

Matrix-assisted laser desorption/ionization (MALDI) time-of-flight (TOF) mass spectrometry (MS) has been presented as a superior method for the detection of microorganisms in body fluid samples (e.g., blood, saliva, pus, etc.) However, the performance of MALDI-TOF MS in routine identification of caries-related *Lactobacillus* isolates from saliva of adult patients with caries has not been determined. In the present study, we introduced a new MALDI-TOF MS system for identification of lactobacilli. Saliva samples were collected from 120 subjects with caries. Bacteria were isolated and cultured, and each isolate was identified by both 16S rRNA sequencing and MALDI-TOF MS. The identification results obtained by MALDI-TOF MS were concordant at the genus level with those of conventional 16S rRNA-based sequencing for 88.6% of lactobacilli (62/70) and 95.5% of non-lactobacilli (21/22). Up to 96 results could be obtained in parallel on a single MALDI target, suggesting that this is a reliable high-throughput approach for routine identification of lactobacilli. However, additional reference strains are necessary to increase the sensitivity and specificity of species-level identification.

## Introduction

Lactobacilli were the first microorganisms implicated in the development of dental caries [1] and have been shown to be associated with the dental carious process [2]. The *Lactobacillus* count represents the number of lactobacilli present in 1 mL of saliva (CFU/mL), and is used to determine the efficiency of dietetic measures or to evaluate caries risk. It can be used alone or in combination with other associated parameters [3], [4]. Granath et al. [5] suggested that the saliva count of lactobacilli is a better criterion than that of *Streptococcus mutans* as the former count is strongly correlated with caries [6]–[12]. Specifically, a higher decay missing fill (DMF) index is associated with a greater number of children harboring a high *Lactobacillus* count [13], [14].

Although there is a strong association between lactobacilli and caries, little is known regarding the relationship at the species level because of difficulties in identifying *Lactobacillus* species by conventional methods [15].

Efforts have been made to develop simple and rapid methods for discriminating *Lactobacillus* species, and the advent of novel molecular tools has facilitated identification.

Matrix-assisted laser desorption/ionization time-of-flight mass spectrometry (MALDI-TOF MS) is a rapid, accurate, and cost-effective method used to identify microorganisms [16]–[18]. Although MALDI-TOF MS was introduced into routine microbiological diagnoses with marked success in many fields [19]–[22], there is still room for improvement in identification of lactobacilli, especially in salivary samples as the *Lactobacillus* strains found in saliva are phenotypically very similar [23]. Most recent studies regarding identification of microorganisms by MALDI-TOF MS were based on the Bruker system [24], [25], which has already been commercially developed for clinical application. However, differing from these evaluative studies, we introduced a new MALDI-TOF MS system for identification of lactobacilli. We provided further first-hand information regarding the identification process, such as database establishment and principles of identification, to facilitate future improvement in identification of lactobacilli or other species.

Little is known regarding the oral *Lactobacillus* species associated with adult caries. To gain a greater understanding of the roles of various lactobacilli in the development of dental caries in adults, we assessed the capability of MALDI-TOF MS to identify lactobacilli isolated from the saliva of adult patients with caries.

## Materials and Methods

### Study population

The study population consisted of 120 adults, 18 – 75 years old, with dental caries. Between April and June 2013, patients were recruited from the Department of Endodontics of the Peking University School and Hospital of Stomatology, and 30 subjects from the Department of Endodontics of Beijing Stomatological Hospital Capital Medical University.

Three clinicians performed the clinical examinations. Each subject was examined according to WHO standard methods and criteria [WHO, 1997]. Non-stimulated saliva samples were collected from each subject. The Institutional Review Board of Peking University School and Hospital of Stomatology approved the study protocol (approval number PKUSSIRB-2013021). And participants provided their written informed consent to participate in this study.

### Isolation and identification of lactobacilli

For each sample, a 60-µL aliquot of saliva was spread on selective medium that promotes the growth of lactobacilli while suppressing growth of other bacteria (Rogosa agar; Hope Bio-Technology Co., Qingdao, Shandong, China). After incubation in a 5% CO_2_ incubator at 37°C for 48 h, a single colony with the most predominate morphology was picked and sub-cultured for 24 h on Rogosa agar under the same conditions. Then, a colony from the sub-culture was analyzed by 16S rRNA sequencing and another was examined by MALDI-TOF MS for identification.

For 16S rRNA sequencing, the primers 16S-27f (AGAGTTTGATCCTGGCTCA) and 16S-1492r (GGTTACCTTGTTACGACTT) were used for PCR. All sequencing reactions were performed using a standard thermocycler with 35 cycles of denaturation (30 s at 95°C), annealing (60 s at 60°C), and extension (1 min 45 s at 72°C). Sequencing was performed by BGI Tech Company (Beijing, China) and the obtained sequences were compared with those in the National Center of Biotechnology Information (NCBI) database by BLAST searching.

### Mass mapping by MALDI-TOF MS

Bacterial extracts for MS were prepared as follows. A colony was transferred into a 1.5-mL centrifuge tube using an inoculation loop. A 300-µL aliquot of deionized water and 900 µL of absolute ethanol were then added, and the mixture was vortexed for 1 min prior to centrifugation (16200 × *g*, 10 min).

Following centrifugation, the supernatant was removed, and the bacterial pellet was then air-dried, resuspended in 50 µL of 70% formic acid, and vortexed. Then 50 µL of 100% acetonitrile was added, and the resulting mixture was vortexed and centrifuged at 16200× g for 5 min. Aliquots of 1 µL of the supernatant were spotted onto a ground steel target in triplicate (Bioyong, Beijing, China), and air-dried for 10 min. Each spot was overlaid with 1 µL of matrix solution (α-cyano-4-hydroxycinnamic acid in 50% acetonitrile/0.1% trifluoroacetic acid), and allowed to co-crystallize for 1 min at room temperature. Spectra were acquired using a Clin-ToF mass spectrometer (Bioyong,Beijing,China) in linear positive mode (laser frequency, 20 Hz; acceleration voltage, 20 kV; pulser voltage, 1.9 kV; Einzel voltage 5 kV; delayed ext. focus mass, 10000; mass range, 2000 – 13000 Da).

The composite profile was analyzed using BioExplorer 1.0 software (Bioyong) with default parameter settings. The following reference strains were used to establish a database: *Lactobacillus acidophilus* ATCC 4356, *Lactobacillus casei* ATCC 334, *Lactobacillus fermentum* ATCC 14931, *Lactobacillus oris* ATCC 49062, *Lactobacillus plantarum* ATCC 8014, *Lactobacillus rhamnosus* ATCC 7469, and *Lactobacillus salivarius* ATCC 11741. The main spectra of each reference strain were obtained using the “training” process. Briefly, each reference strain was spotted in 72 duplicate samples, and 72 spectra were obtained. The peaks with the least detected frequency (e.g., < 10%) were excluded. The peak list of the main spectra and the frequency of each peak were obtained.

Unknown spectra were identified within BioTyper (Bioyong, Beijing, China) by comparison with reference spectra (MSPs). The identification process starts with preprocessing of unknown spectra and generation of peak lists. The peak lists of unknown spectra are then compared and aligned with those of MSPs. In the first step, unknown spectra are calibrated to the MSPs. For this calibration an initial mass error can be selected within the identification parameters (parameter Max. Mass Error of the Raw Spectrum). BioTyper attempts to align the highest peaks of each spectrum within the borders of the initial mass error. While keeping quadratic calibration constant, peaks were aligned and spectra were calibrated. The goal was to reach the highest grade of homology.

In the second step, the matching of unknown spectra to the MSPs was evaluated based on dedicated score values. For this, peak information of the MSPs was transformed to a maximum accessible score (point) value. The peak frequency corresponds to the occurrence of the respective peak within the spectra used for generation of the MSPs. A final score value for the unknown spectrum is calculated according to the discrepancy between the unknown spectrum and reference spectrum, the number of matched spectrum and the frequence of common spectrum. Peaks with lower frequencies show the appropriate scores. Scores of ≥ 10.0 were considered high-confidence identification. For each specimen, three replicates were measured and specimens for which at least two tests scored > 10 were identified as *Lactobacillus*.

## Results

### Isolation and distribution of lactobacilli in caries-positive adults

**Figure 1:**
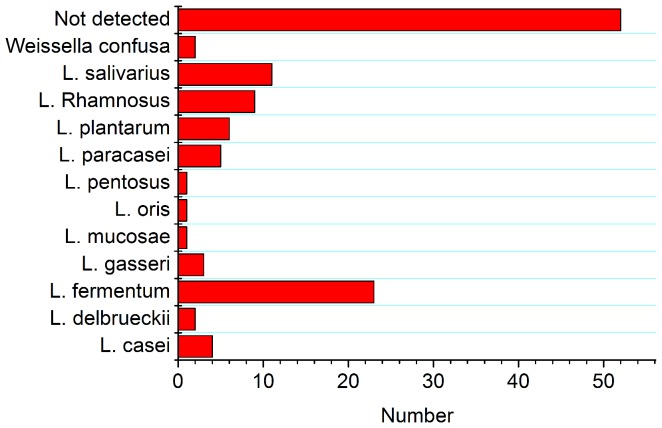
Distribution of *Lactobacillus* species in the saliva of adult patients with caries as determined by 16S rRNA sequencing.

Saliva samples were collected from 120 carious patients, and cultured in Rogosa agar for selection of lactobacilli. Samples from 28 subjects did not grow on Rogosa plates, and 22 of the remaining 92 were non-lactobacilli, such as *Streptococcus*, and were therefore negative isolates. A total of 70 lactobacilli were cultured from 120 saliva samples (58.3%). The distribution of *Lactobacillus* species detected in carious patients is shown in Figure 1; the predominant species found were *L. fermentum*, *L. salivarius*, and *L. rhamnosus*.

### Establishing a detection model using MALDI-TOF MS

**Figure 2:**
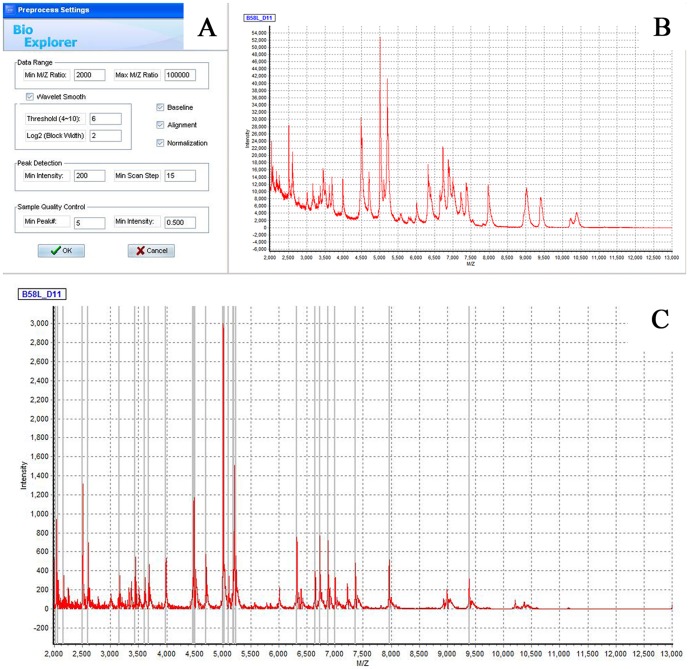
The raw data (MALDI-TOF profile mass spectra) of the sample strain (B) were preprocessed through the default settings (A), and the normalized spectra (C) were obtained.

**Figure 3:**
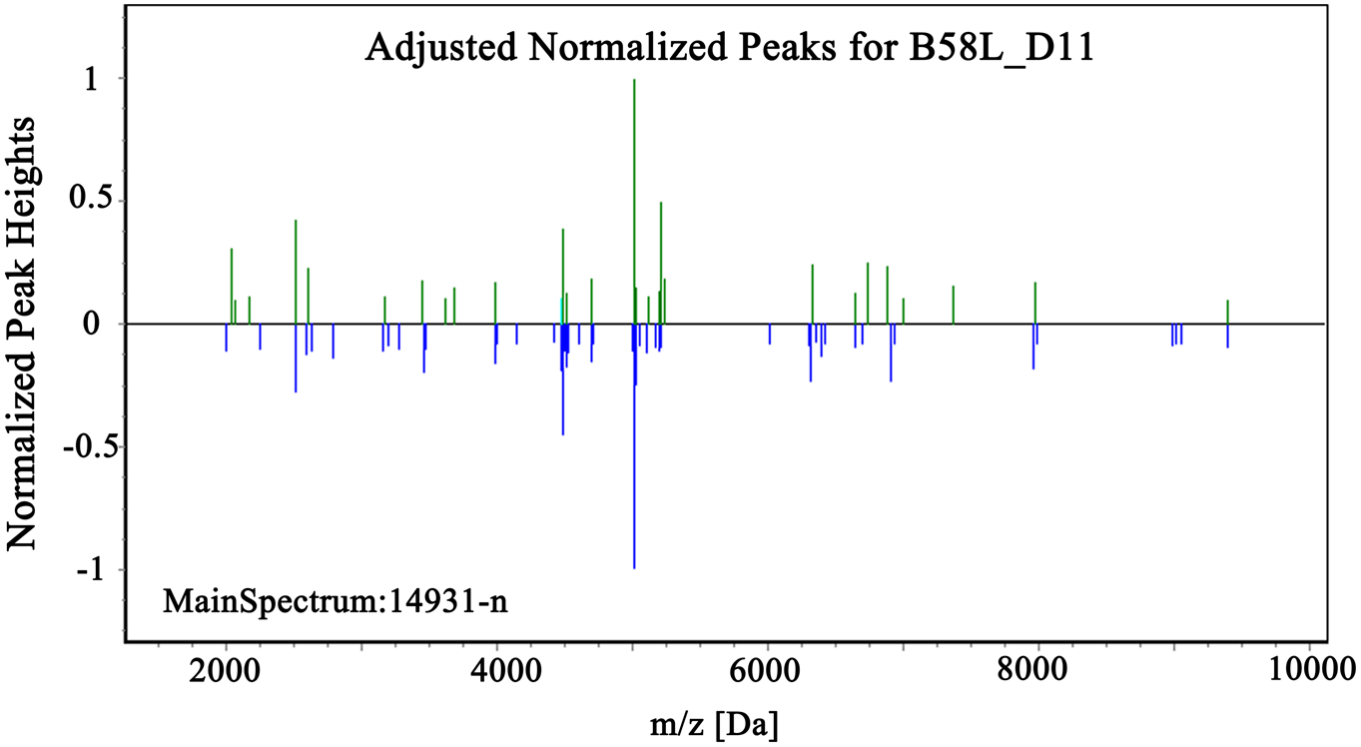
The spectra of the isolated strains (top) were compared with the main spectra of the reference strains (bottom).

Figure S1 shows typical spectra of seven reference strains and one clinical isolate. The majority of the peaks were obtained below a mass/charge (*m/z*) ratio of 8000, and no peaks were observed above an *m/z* of 10000. Spectra are characteristic and differ between species. The raw data were calibrated during the “preprocess” process, including baseline adjustment, spectra denoising, and wavelet smoothing (Fig. 2). Statistical analysis was performed on peak selection (Fig. 2C) using the BioExplorer software. Each sample spectrum was then compared with the main spectra of the reference strains during the “identification” process (Fig. 3). In the graphical output, matching peaks within 2 Da are shown in green, those within 5 Da are in yellow, and non-matching peaks are shown in red. From the cumulative score value of the unknown spectrum and the maximum score of the MSP, a final score value for the unknown spectrum is calculated (Table. 1). A higher score is given when the sample spectrum has more common peaks with the higher frequency reference spectra. The result would be considered correct if identical to the 16S rRNA sequencing result.

**Table 1:** The information from, and frequency of, coincident peaks were converted into scores (see column 1000*pro).

Report	Detected Species	Act Sc	Max Sc	Rel Score	PN k	PN b	PN m	Rel P-Nu…	I-Corr.	1000*Pro
B58L_D10	14931-n	1111	2272	0.49	7	6	27	0.37	0.83	151
	ATCC4356	350	3192	0.11	1	3	27	0.09	1.00	10
	ATCC8014	136	2500	0.05	0	6	27	0.11	0.97	5
	ATCC11741	226	3692	0.06	1	2	27	0.07	1.00	4
	7469-n	71	2412	0.03	0	4	27	0.07	0.16	0
	49062-n	100	1835	0.05	1	2	27	0.07	0.00	0
	ATCC334	0	2348	0.00	1	0	27	0.04	0.00	0
B58L_D11	14931-n	1220	2272	0.54	6	8	28	0.36	0.88	168
	ATCC8014	136	2500	0.05	0	6	28	0.11	0.97	5
	ATCC4356	250	3192	0.08	1	2	28	0.07	0.98	5
	ATCC11741	126	3692	0.03	0	2	28	0.04	1.00	1
	7469-n	71	2412	0.03	0	4	28	0.07	0.18	0
	49062-n	100	1835	0.05	1	2	28	0.07	0.00	0
	ATCC334	0	2348	0.00	0	1	28	0.02	0.00	0
B58L_D12	14931-n	1129	2272	0.50	6	8	25	0.40	0.80	159
	49062-n	137	1835	0.07	2	1	25	0.10	1.00	7
	ATCC4356	196	3192	0.06	1	2	25	0.08	0.97	4
	ATCC11741	182	3692	0.05	1	2	25	0.08	0.81	3
	ATCC8014	164	2500	0.07	0	4	25	0.08	0.15	0
	7469-n	51	2412	0.02	0	2	25	0.04	0.00	0
	ATCC334	0	2348	0.00	0	0	25	0.00	0.00	0

### Identification of lactobacilli by MALDI-TOF MS

Among all of the samples, 83 of 92 (90.2%) isolates were identified correctly using MALDI-TOF MS. Specifically, 62 of 70 (88.6%) positive isolates and 21 of 22 (95.5%) negative isolates were identified correctly at the genus level (Table 2). Table 3 shows the results of species identification. For species with standard spectra in the database, the identification was correct at the species level. For example, the identification rates of *L. fermentum*, *L. rhamnosus*, and *L. plantarum* were 80% (20/25), 75% (6/8), and 100% (7/7), respectively. However, the correct rate was only 36.4% (4/11) for *L. salivarius* at the species level.

**Table 2:** Identification of lactobacillus in 92 clinical isolates obtained using MALDI-TOF MS compared with partial 16S rRNA gene sequence-based identification.

	16S rRNA sequencing results		Total
	Positive	Negative	
MALDI-TOF MS			
Positive	62	1	63
Negative	8	21	29
Total	70	22	92

**Table 3:** Clinical isolates identified by MALDI-TOF MS.

	No. of strains	No. identified correctly	
		Genus level	Species level
*Lactobacillus casei*	4	3	3
*Lactobacillus delbrueckii*	2	1	0
*Lactobacillus fermentum*	25	23	20
*Lactobacillus gasseri*	3	2	0
*Lactobacillus mucosae*	1	1	0
*Lactobacillus oris*	1	1	1
*Lactobacillus paracasei*	5	5	0
*Lactobacillus pentosus*	1	0	0
*Lactobacillus plantarum*	7	7	7
*Lactobacillus Rhamnosus*	8	8	6
*Lactobacillus salivarius*	11	9	4
*Weissella confusa*	1	1	0

## Discussion

The effectiveness of MALDI-TOF MS for identification of bacterial species was demonstrated previously [18], [21], [26], [27]. In this study, we established assessed the effectiveness of a novel identification method. The MALDI BioExplorer software compares unknown mass spectra with reference data stored in a database by pattern matching. First, the software extracts a list of mass spectrum peaks after smoothing and baseline subtraction. This list of peaks is compared with each entry in the reference database, and the unknown peak in the list is aligned with each main spectrum by a dedicated recalibration algorithm. In addition, correlation of the frequencies of matching peaks is performed. A score of this identification is then calculated based on peak matches and frequency correlations. After comparing the unknown spectrum with all of the main spectra in the database, the score values are summed. In the present study, 90.2% of isolates were identified correctly to the genus level using the MALDI-TOF MS-based method (Table 2). These data were consistent with those obtained using the reference method, and were comparable to the results of previous studies using other MALDI-TOF mass spectrometry systems [25], [28]. These observations suggest that MALDI-TOF MS is a powerful method for the rapid identification of lactobacilli in human saliva. The *Lactobacillus* genus includes more than 80 species, ∼20 of which have been found in the oral cavity [29]. We selected seven species that are closely related to dental caries; i.e., *L. fermentum*, *L. rhamnosus*, *L. plantarum*, *L. casei*, and *L. salivarius* [15], [25], [30]. The precise identification of these lactobacilli at the species level was effective for all except *L. salivarius*, which may have been due to the low quality of the standard spectrum of *L. salivarius* (Fig. S1). To improve the database, the experimental process should be further optimized. Furthermore, to limit the data pool, not all colonies from the saliva samples can be identified at the species level. Therefore, it is important to enrich the information content of the data pool. Meanwhile, more *Lactobacillus* colonies in the saliva samples may be needed to confirm the effectiveness of the identification. However, the establishment of a database and identification were shown to be feasible.

Saliva is a clinically informative fluid that is useful for novel approaches to prognosis, clinical diagnosis, and the monitoring and management of patients with oral and systemic diseases, because it contains specific soluble biological markers that allow early disease detection [31], [32]. Positive correlations between salivary caries-related findings and caries prevalence have been established in groups of children [33] and adolescents [34], and the removal and restoration of caries regions would reduce the numbers of salivary lactobacilli [35], [36]. The dominant bacterial species in the saliva of a carious patient may serve as an indicator for predicting caries susceptibility, and may also facilitate identification of subjects at a high risk of caries. Determination of bacterial markers that could serve as indicators for predicting caries susceptibility would require a rapid and efficient method of screening carious patients [15]; MALDI-TOF MS is one such method. In this study, we confirmed the potential of MALDI-TOF MS for caries-related bacterial detection. The model is well suited to detection of lactobacilli, and would be appropriate for detection of other bacteria using different databases. Furthermore, it will facilitate investigation of the prevalence of various *Lactobacillus* species in the oral cavity and in relation to the caries frequencies of different age groups.

## Supporting Information

Figure S1 **Comparison of seven Lactobacillus reference species (** ***L. acidophilus*** **ATCC 4356,** ***L. casei*** **ATCC 334,** ***L. fermentum*** **ATCC 14931,** ***L. oris*** **ATCC 49062,** ***L. plantarum*** **ATCC 8014,** ***L. rhamnosus*** **ATCC 7469, and** ***L. salivarius*** **ATCC 11741) with a Lactobacillus isolate derived from carious subjects’ saliva.**
(PDF)
