# Supplementary material for: Identification of Lactobacillus from the Saliva of Adult Patients with Caries Using Matrix-Assisted Laser Desorption/Ionization Time-Of-Flight Mass Spectrometry
Source: PLoS One. 2014 Aug 28;9(8):e106185. doi: 10.1371/journal.pone.0106185 (PMC4148440; doi:10.1371/journal.pone.0106185)
Supplement: Figure S1 — Comparison of seven Lactobacillus reference species ( L. acidophilus ATCC 4356, L. casei ATCC 334, L. fermentum ATCC 14931, L. oris ATCC 49062, L. plantarum ATCC 8014, L. rhamnosus ATCC 7469, and L. salivarius ATCC 11741) with a Lactobacillus isolate derived from carious subjects’ saliva. (PDF) [file pone.0106185.s001.pdf]

Supplementary

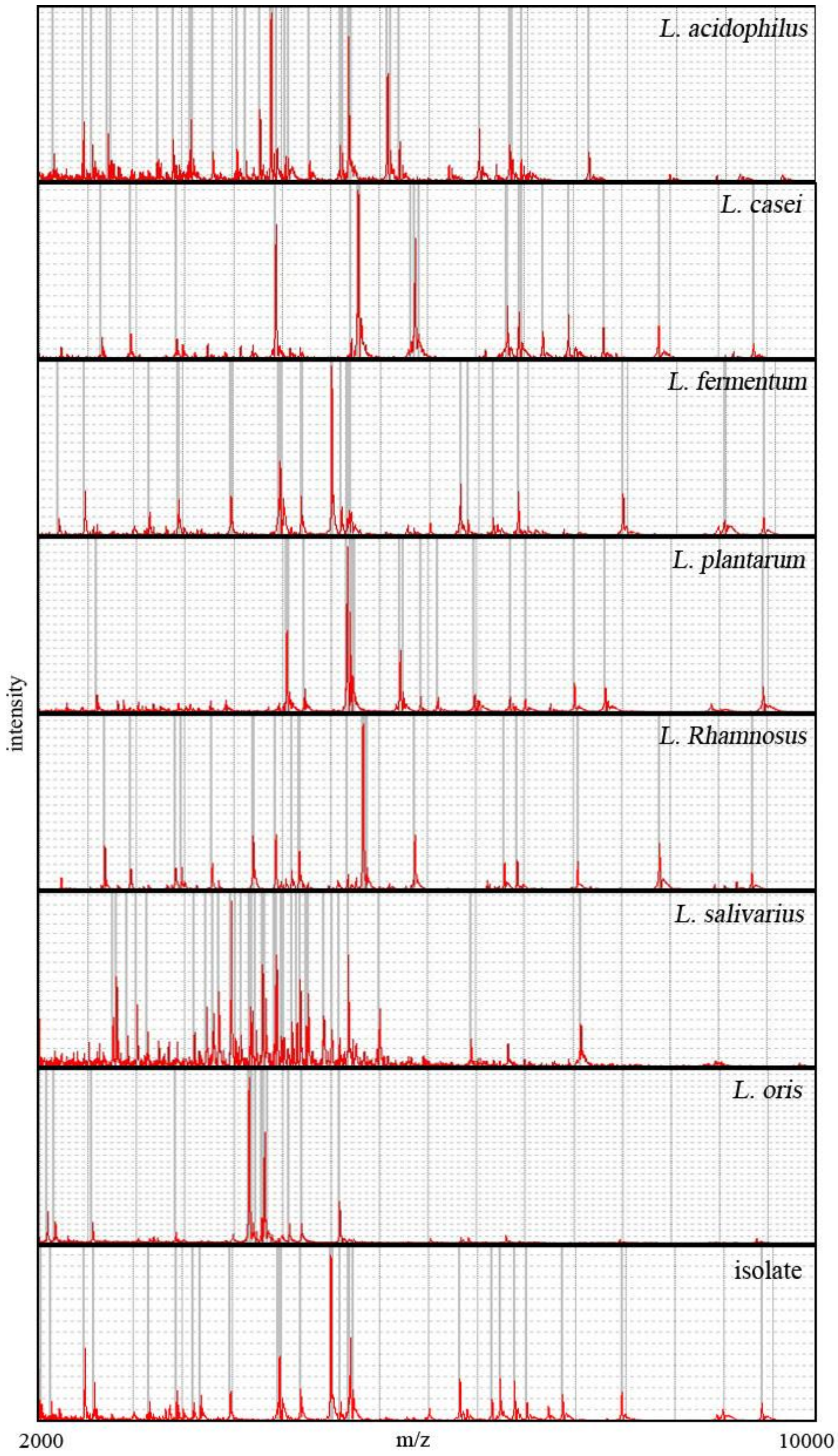

L. acidophilus
L. casei
L. fermentum
L. plantarum
L. Rhamnosus
L. salivarius
L. oris
isolate
intensity
2000
m/z
10000

Figure S1: Comparison of seven Lactobacillus reference species (*L. acidophilus* ATCC 4356, *L. casei* ATCC 334, *L. fermentum* ATCC 14931, *L. oris* ATCC 49062, *L. plantarum* ATCC 8014, *L. rhamnosus* ATCC 7469, and *L. salivarius* ATCC 11741) with a Lactobacillus isolate derived from carious subjects' saliva.
